# Integrated multi-locus genome-wide association studies and transcriptome analysis for seed yield and yield-related traits in *Brassica napus*

**DOI:** 10.3389/fpls.2023.1153000

**Published:** 2023-04-14

**Authors:** Cuiping Zhang, Ruolin Gong, Hua Zhong, Chunyan Dai, Ru Zhang, Jungang Dong, Yangsheng Li, Shuai Liu, Jihong Hu

**Affiliations:** ^1^ State Key Laboratory of Crop Stress Biology for Arid Areas, College of Agronomy, Northwest A&F University, Yangling, China; ^2^ Cancer Epidemiology Division, Population Sciences in the Pacific Program, University of Hawaii at Manoa, Honolulu, HI, United States; ^3^ State Key Laboratory of Hybrid Rice, College of Life Sciences, Wuhan University, Wuhan, China

**Keywords:** rapeseed, yield, seed weight, multi-locus GWAS, candidate gene, RNA-seq

## Abstract

Rapeseed (*Brassica napus* L.), the third largest oil crop, is an important source of vegetable oil and biofuel for the world. Although the breeding and yield has been improved, rapeseed still has the lowest yield compared with other major crops. Thus, increasing rapeseed yield is essential for the high demand of vegetable oil and high-quality protein for live stocks. Silique number per plant (SN), seed per pod (SP), and 1000-seed weight (SW) are the three important factors for seed yield in rapeseed. Some yield-related traits, including plant height (PH), flowering time (FT), primary branch number (BN) and silique number per inflorescence (SI) also affect the yield per plant (YP). Using six multi-locus genome-wide association study (ML-GWAS) approaches, a total of 908 yield-related quantitative trait nucleotides (QTNs) were identified in a panel consisting of 403 rapeseed core accessions based on whole-genome sequencing. Integration of ML-GWAS with transcriptome analysis, 79 candidate genes, including *BnaA09g39790D* (*RNA helicase*), *BnaA09g39950D* (*Lipase*) and *BnaC09g25980D* (*SWEET7*), were further identified and twelve genes were validated by qRT-PCRs to affect the SW or SP in rapeseed. The distribution of superior alleles from nineteen stable QTNs in 20 elite rapeseed accessions suggested that the high-yielding accessions contained more superior alleles. These results would contribute to a further understanding of the genetic basis of yield-related traits and could be used for crop improvement in *B. napus*.

## Introduction

*Brassica napus* (*B. napus*, AACC, 2n = 38) is one of the most important oilseed crops worldwide as vegetable oil, animal feed and biofuel (Lu et al., 2019; Song et al., 2020). However, the deteriorating environment and lack of arable land make the yield of rapeseed insufficient to support the demand. Therefore, increasing rapeseed yields is a research priority for rapeseed breeders to meet the future demand of oilseed rape production. The yield of rapeseed is a complex quantitative trait and mainly determined by three yield-component traits, including 1000-seed weight (SW), silique number per plant (SN) and seed per pod (SP) (Zhao et al., 2016; Lu et al., 2017). Seed yield is also influenced by yield-related traits such as plant height (PH), flowering time (FT), primary branch number (BN), length of main inflorescence, and silique number of main inflorescence (SI) in rapeseed (Shi et al., 2009; Zhao et al., 2016). There is a correlation among these yield traits, and they interact with each other to jointly determine the rapeseed yield. In addition, the relationships between these traits are intricate, for example, SW was positively correlated with yield per plant, plant height and length of main inflorescence, while it was negatively correlated with seed number per pod and flowering time. Seed size affects the SW and SP, which is of great value for crop improvement in rapeseed (Li et al., 2019a). Thus, dissecting the genetic basis and molecular mechanism of yield traits will facilitate and accelerate breeding programs for yield in rapeseed.

Rapeseed yield component and related traits are all complex quantitative traits governed by multiple genes. In previous studies, some loci or genes for yield traits in rapeseed were identified using quantitative trait locus (QTL) mapping and map-based cloning (Shi et al., 2009; Li et al., 2015; Liu et al., 2015; Li et al., 2019b; Shi et al., 2019; Wang et al., 2021a). Zhou et al. (2014) mapped 736 QTLs associated with the yield in the A and C subgenomes of *B. napus*, which were distributed over 19 chromosomes, mostly located on A03. Raboanatahiry et al. (2018) detected 972 QTLs associated with seed yield and yield-related traits in *B. napus*, identifying 147 potential candidate genes that could affect nine different traits. With the development of high-density customized single nucleotide polymorphism (SNPs), genome wide association study (GWAS) has become a powerful tool for deciphering the genetic architecture of complex quantitative traits (Lu et al., 2017; Zhong et al., 2021a). Based on 33,186 genomic SNPs from the 60 K *Brassica* Illumina Infinium SNP array, a new QTL was fine mapped onto chromosome C03 in *B. napus* and a gene controlling the branching number phenotype was identified (He et al., 2017). Integrated GWAS and transcriptome analysis, auxin-related genes were identified to associate with leaf petiole angle at the seedling stage in *B. napus* (Hu et al., 2021a). And stable QTLs localized on chromosomes A07, A09, and C08 were identified for silique length (SL) using GWAS combined with RNA-seq (Wang et al., 2021a). With the advance of next-generation sequencing (NGS) technology, mega-level SNPs or genetic variations *via* whole genome resequencing in population could detect more loci for the yield traits in crops (Zhang et al., 2022a). Using 10,658 high-quality SNP markers, a total of 497 SNPs were detected to associate with yield-related traits in *B. napus* (Zhang et al., 2022b). Based on high-quality 670,028 SNPs, GWASs were conducted using multi-locus random mixed linear model for 11 important traits in rapeseed (Lu et al., 2019). Using a nested association mapping population, SNP-GWAS, and presence and absence variation (PAV)-GWAS identified loci and structural variations for silique length, SW and FT (Song et al., 2020). Furthermore, 56 agronomically traits, including plant architecture and yield traits were examined using whole-genome resequencing in 403 diverse rapeseed accessions by GWAS and identified 26 loci associated with SW and silique length (Hu et al., 2022). Most of these GWAS studies used the single-locus GWAS (SL-GWAS) methods, such as the general linear model (GLM), the mixed linear model (MLM), efficient mixed-model association eXpedited (EMMAX), and factored spectrally transformed linear mixed models (FaST-LMM) (Zhong et al., 2021b). However, the SL-GWAS methods rule out many significant loci, including minor effect loci due to their highly stringent Bonferroni correction (Wang et al., 2016).

Recently, multi-locus GWAS (ML-GWAS) methodologies, including FASTmrEMMA, ISIS EM-BLASSO, mrMLM, FASTmrMLM, pLARmEB, pKWmEB, were developed as an effective approach for association analysis (Cui et al., 2018; Zhang et al., 2020). And various combinations of ML-GWAS methods have proven to be significantly effective in controlling false positive rates (Misra et al., 2017; Zhong et al., 2021c). Thus, ML-GWAS has been used to discover novel quantitative trait nucleotides (QTNs) in many crops. In wheat, five ML-GWAS models were utilized to successfully identify new QTL for yield-related traits in 272 local Chinese wheat landraces based on 172,711 SNPs (Lin et al., 2021). ML-GWAS of 144 maize inbred lines genotyped with 43,427 SNPs identified a large number of significant QTNs and 40 candidate genes associated with the regenerative capacity of the embryonic callus (Ma et al., 2018). In soybean, 129 significant QTNs related to protein content were identified by five ML-GWAS methods, and 8 candidate genes were predicted to be involved in protein synthesis and metabolism (Zhang et al., 2018). Using ML-GWAS, 74 significant QTN hotspots have been identified to associate with five yield-related traits in rice (Zhong et al., 2021c). However, ML-GWAS methods for yield-related traits in rapeseed have not yet been performed, especially based on the whole genome resequencing data. Given the efficiency and various models of ML-GWAS, more novel loci and candidate genes could be identified to associate with yield traits.

In the present study, we aimed to identify novel loci for seed yield and yield-related traits in rapeseed. Six ML-GWAS approaches were used to determine novel QTNs based on high-quality SNPs in 403 rapeseed accessions for eight yield traits in three environments. And we also analyzed the significant QTNs and pinpointed multiple candidate causal genes for the QTNs. The candidate genes and elite alleles identified with yield traits will provide an insight into further exploration of the genetic architecture of yield traits in rapeseed and genetic improvement of rapeseed.

## Materials and methods

### Plant materials and phenotype evaluation

A highly diverse natural population consisting of 403 core rapeseed germplasms were used as previous study (Hu et al., 2022). The set of rapeseed accessions includes spring (102), semi-winter (179) and winter (129) types from China, Germany, France, Canada, Japan, USA and other countries. And phenotyping of the 403 accessions were evaluated under three environments: E1, Yangluo (30.38° N, 114.50° E) in 2013, E2, Nanchang (28.37° N, 116.27° E) in 2014, and E3, Wuhan (30.58° N, 113.68° E) in 2016, which were download from https://www.cgris.net/rapedata/ or http://brassicanapusdata.cn/. The yield traits including SI, SN, SP, SW, and YP as well as three yield-related traits, such as PH, BN, and FT were measured according to the measurement standards (Chen et al., 2014; Li et al., 2016). The large seed size accession R01 and small seed size accession R56 were grown in a greenhouse (light/dark 16/8 h photoperiod and 25°C/20°C day/night temperature) in Northwest Agriculture and Forestry University, Yangling, Shaanxi, China.

### Genotyping data processing

Whole genome resequencing was performed on the Illumina HiSeq 4000 platform with 150 bp paired-end and download from NCBI (PRJNA416679) (Hu et al., 2022). GATK (v.3.3) was used for SNP calling and then excluded SNP calling errors, retaining only high-quality SNPs (minor allele frequency ≥ 0.05, miss ≤ 0.2 and sequencing depth ≥ 6) for subsequent analysis (McKenna et al., 2010). These SNPs were processed by PLINK 1.9 with parameter –maf 0.05 –geno 0.05, –snps-only and imputed by Beagele 5.0 (Browning et al., 2018; Zhong et al., 2021c). Finally, a total of 7,531,945 high-quality SNPs were employed for GWAS analysis.

### Multi locus-GWAS analysis

Six ML-GWAS methods, including the mrMLM, FASTmrMLM, FASTmrEMMA, pLARmEB, pKWmEB, and ISIS EM-BLASSO, were used to identify significant QTN. All six ML-GWAS approaches are implemented in R package “mrMLM” (https://cran.r-project.org/web/packages/mrMLM/index.html) (Wang et al., 2016). Default values were used for all parameters and a threshold of logarithm of odds (LOD ≥ 3 or *P* ≤ 0.0002) was chosen to examine the association between markers and yield-related traits (Zhang et al., 2019). Principal component analysis and kinship matrices were used in all methods. The R package CMplot (https://github.com/yinliLin/R-CMplot) was employed to visualize Manhattan and QQ plots of GWAS. Using the Tassel 5.2 tool (Bradbury et al., 2007), LDs between SNPs were estimated as the squared correlation coefficient (*R* ^2^) of alleles, and *R* ^2^ values were calculated within a 0 to 10 cM window. The phenotypic-effect value of allelic variation for each trait was calculated by the phenotypic data across the 403 accessions. The relative phenotypic data were visualized with box plots using the R 4.2.1 software.

### Identification of candidate genes

The putative candidate loci were identified by at least two different GWAS methods. Candidate genes were predicted from the upstream or downstream 200 kb region of stable QTL loci using the ‘Darmor-*bzh*’ reference genome (https://www.genoscope.cns.fr/brassicanapus) (Chalhoub et al., 2014). Then the candidate genes were further annotated on NCBI (https://www.ncbi.nlm.nih.gov/) and annotated for *Arabidopsis* homologous genes by BLAST analysis (Hu et al., 2021a). Haplotype analysis of the QTN association regions across the 403 rapeseed accessions was conducted by Haploview v4.2 (Barrett et al., 2005).

### Transcriptome analysis

Total RNA was extracted from developing siliques (include seeds) of two extremely SW accessions R01 (large seed) and R56 (small seed) for RNA-seq at two weeks after pollination (2 WAP), three weeks after pollination (3 WAP), and four weeks after pollination (4 WAP). Illumina’s NEBNext^®^ UltraTM RNA Library Preparation Kit was used to construct libraries and quality control was checked by Agilent 2100 Bioanalyzer System. Clean reads after filtering the raw reads were mapped to the “Darmor-*bzh*” reference genome (https://www.genoscope.cns.fr/brassicanapus) using HISAT2 software (Chalhoub et al., 2014; Kim et al., 2015). Gene expression levels were normalized using the FPKM (fragments per kilobase per million reads) values by StringTie (Pertea et al., 2015). Differential expression analysis between the sample pairs in two rapeseed accessions was conducted by DESeq2 (Love et al., 2014). Differentially expressed genes (DEGs) were determined with false discovery rate (FDR) < 0.05 and |log2 (fold change)| ≥ 1. GO (Gene ontology) and KEGG (Kyoto Encyclopedia of Genes and Genomes) pathway enrichment analysis of the DEGs were performed using AgiGO 2.0 and KOBAS3.0, respectively (Tian et al., 2017; Bu et al., 2021).

### Candidate gene expression analysis

Candidate genes were predicted based on the ML-GWAS and transcriptome analysis, and were further validated by quantitative real-time PCR (qRT-PCR). Briefly, 1μg of total RNA was used for RNA-seq and cDNA synthesis was performed using HiScript^®^Q RT SuperMix (Vazyme, China). Data collection was performed in QuantStudio™ real-time PCR software (Thermo Fisher Scientific, Waltham, MA, USA). All the primers are listed in **Supplementary Table S1**. Data were normalized by the internal control gene *BnACTIN* (*BnaA03g55890D*) and relative expression levels were calculated using a 2^-ΔΔCT^ analysis method (Livak and Schmittgen, 2001).

## Results

### Phenotypic evaluation for yield traits

**Figure 1:**
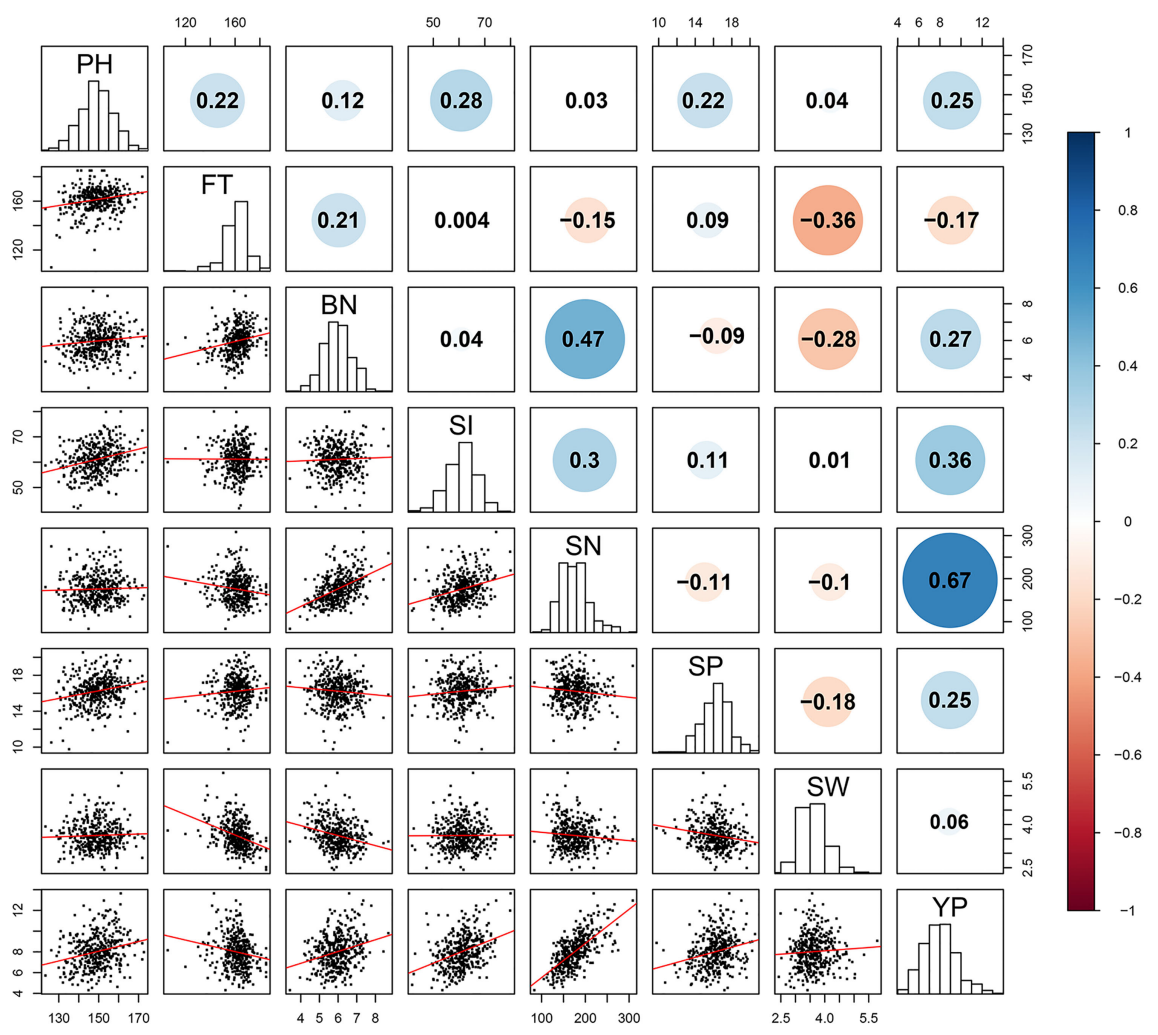
Pairwise Pearson correlation among the eight yield traits in *B.napus*. The upper diagonal represents the Pearson correlation coefficient (PCC) between every two traits (positive numbers represent positive correlation, negative numbers represent negative correlation). The diagonal histogram represents the distribution of each trait, and the lower diagonal represents the linear regression statistics between each two traits. Blue for positive correlation, red for negative correlation. The size of the circle represents the absolute magnitude of the PCC.

Phenotypic values for eight yield traits of rapeseed in three environments, including PH, FT, BN, SI, SN, SP, SW, and YP were used to determine whether significant phenotypic differences existed in these traits (**Supplementary Figure S1**). The phenotypic assessments revealed a wide range of variation between the different accessions, with the frequency distribution of all traits approximating a normal distribution (**Figure 1**). In addition, we noted that yield-related traits were differentially affected by environments, with SW and SP remaining relatively stable across environments, while PH and BN were more variable. Taken together, the extent of available variation for the different traits suggested that the set of rapeseed accessions are suitable for GWAS analysis.

To uncover the relationships of the different yield traits, Pearson correlation coefficient (PCC) was used to assess correlations between pairs of traits in the eight yield-related traits (**Figure 1**). SN and YP were highly significantly and positively correlated (PCC=0.67), indicating that the YP was greatly determined by the SN. In addition, there were also significant positive correlations between BN and SN, SI and YP, and SI and SN, with their PCCs of 0.47, 0.36 and 0.3, respectively, while SW was negatively correlated with both FT (PCC = –0.36) and BN (PCC = –0.28). These results suggest that there is an intricate relationship between yield-related traits and they play important roles in regulating oilseed rape yield in a coordinated manner.

### Genome-wide association mapping for yield traits

A total of 908 QTNs for eight yield traits were identified using at least two of the six ML-GWAS methods, namely FASTmrEMMA, FASTmrMLM, ISIS EM-BLASSO, mrMLM, pKWmEB, and pLARmEB (**Supplementary Table S2**). QTNs with LOD scores > 3.0 were considered significant trait-related QTNs. The highest number of QTNs for SN was identified to be 127, followed by SI with 126 and the remaining 118, 96, 110, 115, 106, and 110, were associated with PH, FT, BN, SP, SW, and YP, respectively (**Supplementary Table S2**). The number of QTNs detected by multiple methods varied between environments, with higher numbers found in WH16 and NC14, 435 and 431 respectively, compared to 404 in YL13 (**Supplementary Figure S2**). The most abundant QTNs for SI were found in both YL13 and WH16, with 76 and 84 each, while the greatest number of QTNs for SN were identified in NC15. FASTmrMLM identified the highest number of QTNs (320), while FASTmrEMMA found only 20, with other models identifying numbers in the range of 151 ~ 273 (**Supplementary Figure S2**). In addition, we found that the results identified by pKWEmEB and pLARmEB were consistent with each other.

**Figure 2:**
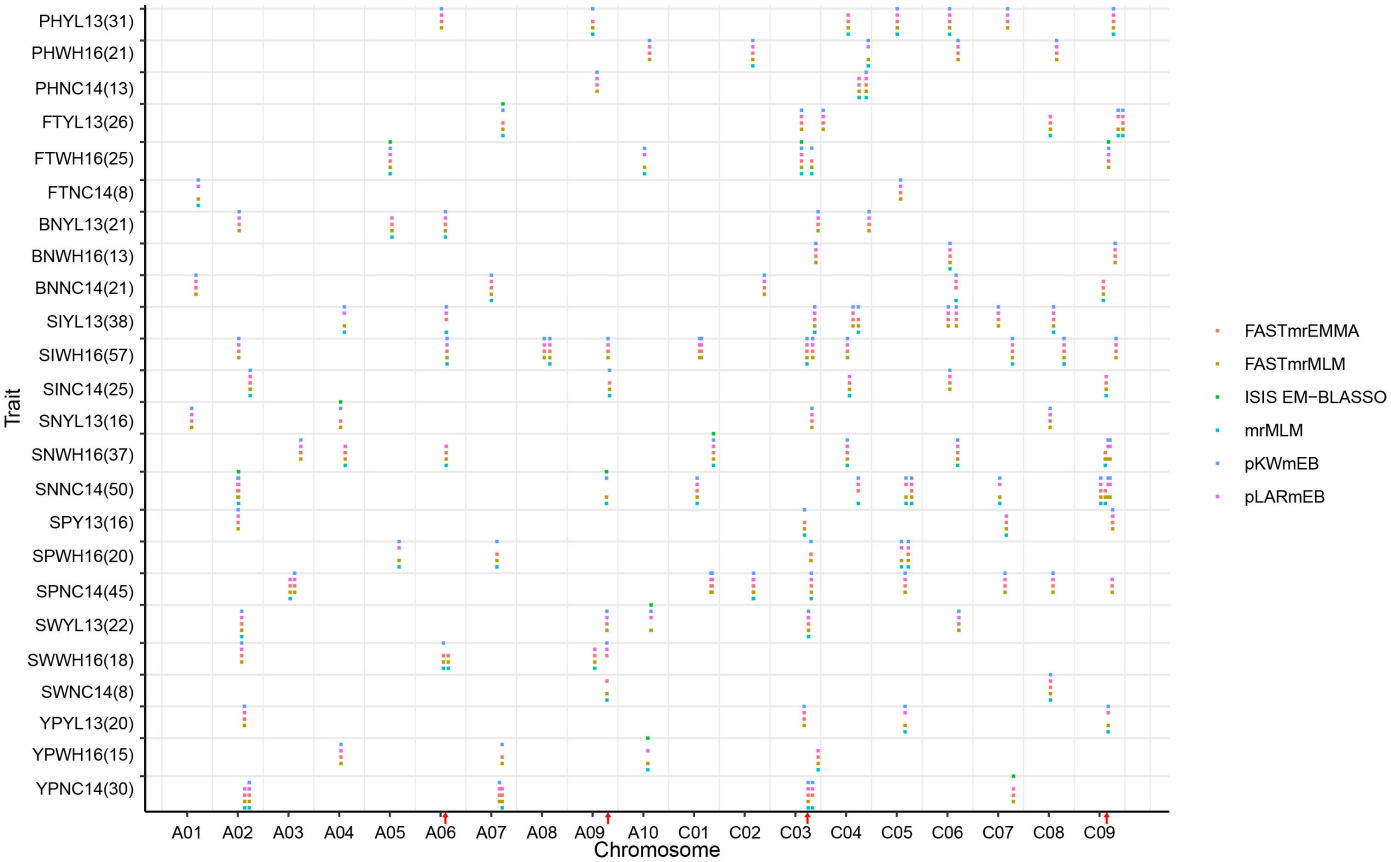
Chromosomal distribution of QTNs for eight yield traits identified by six ML-GWAS methods in the three environments. The horizontal axis indicates genomic locations in chromosomal order and plots significant QTNs according to genomic location. Each row represents a QTN identified by a different ML-GWAS methods. PH, plant height; FT, flowering time; BN, primary branch number; SI, Silique number of main inflorescence; SN, Silique number per plant; SP, seed per pod; SW, 1000-seed weight, YP, yield per plant. Three different environments, YL13, Yangluo 2013; NC14, Nanchang 2014; WH16, Wuhan 2016. The red arrows show the QTN hotspots.

We further analyzed the common QTNs that were co-identified in at least four ML-GWAS approaches (**Figure 2**). Through the combination of different methods, a total of 596 QTNs were identified, of which NC14 was the most, with 236 QTNs, while YL13 was only 154 QTNs. The QTNs associated with SI became the most numerous with 120, the rest being 65, 59, 103, 103, 81, 48, and 65, associated with PH, FT, BN, SN, SP, SW, and YP, respectively (**Figure 2**). These results showed the diversity of QTNs in different environments or traits, demonstrating the importance of identifying stable QTNs by integration of multiple methods.

### Stable QTNs detected by multi-methods or across environments

In order to obtain reliable results, we further analyzed QTNs shared by at least two models in different environments within the 1 Mb region. A total of 75 significant stable QTNs (or QTN clusters) controlling eight yield-related traits were obtained (**Table 1** and **Supplementary Table S3**). One QTN (C03: 12081167) associated with FT in WH16 was identified simultaneously in six models, explaining phenotypic variation of 0.35 ~ 3.79 and LOD scores ranging from 3.04 ~ 5.72, and near which locus, another QTN (C03: 12024986) was also detected in YL13 (**Table 1**). Six QTNs controlling different traits were detected simultaneously in the five models, with *qSWYL13-A02-1* and *qSWWH16-A02-1* for SW explaining the largest phenotypic variation range of 4.10 ~ 22.24. In addition, all QTNs explained the largest range of phenotypic variation of SW (3.05 ~ 27.33), followed by FT (1.78 ~ 16.40). The number of these QTNs varied considerably in the A and C subgenomes, 23 and 44 respectively, and were more numerous on chromosomes C03, C08, and C09, with 10, 7, and 8, respectively.

**Table 1:** Candidate genes associated with eight yield-related traits identified by multi loci-GWAS in different environments.

Trait	Chr.	Position	LOD	*R* ^2^ (%)	Method	Env.	Candidate genes	Annotation
**PH**	C05	35061843	5.11-9.57	2.48-2.76	2,3,4	E1	BnaC05g35940D	AUX/IAA protein
	C05	36252249	6.96-14.20	4.18-5.15	2,3,4	E2	BnaC05g36140D	Protein phosphatase 2C
							BnaC05g37110D	AP2/ERF domain
	C08	18928273	4.69-7.07	1.15-2.97	2,3,4	E2	BnaC08g13850D	SANT/Myb domain
	C08	18801051	4.14-6.64	1.77-2.50	1,2,3	E3	BnaC08g13910D	Protein kinase
							BnaC08g13990D	bHLH
**FT**	A03	23460059	4.76-9.69	2.07-4.25	2,3,5	E1	BnaA03g44520D	Ribonuclease H2
	A03	22616177	6.56-15.01	3.27-4.66	1,2,5	E3	BnaA03g44830D	Pentatricopeptide repeat
							BnaA03g45650D	MADS-box
	C03	12024986	3.07-10.20	2.22-4.77	2,3,4,5	E1	BnaC03g22140D	ABC transporter
	C03	12081167	3.04-5.72	0.35-3.79	1,2,3,4,5,6	E3	BnaC03g22200D	Expansin
							BnaC03g22280D	Amino acid transporter
	C09	45879981	3.77-7.28	1.99-5.74	1,2,3,4,5	E1	BnaC09g45620D	Nonaspanin (TM9SF)
	C09	45879370	4.36-4.83	2.84-4.57	2,4,5	E2	BnaC09g45880D	Cytochrome P450
							BnaC09g45930D	Zinc finger, RING-type
**BN**	C06	4708955	6.18-8.91	2.11-4.13	2,4,5	E1	BnaC06g03900D	AP2/ERF domain
	C06	4978698	5.22-7.61	2.02-3.11	1,2,3,4,5	E3	BnaC06g04200D	F-box domain, cyclin-like
							BnaC06g04380D	bHLH
	C08	24114714	3.37-8.73	2.17-4.08	2,4,5	E2	BnaC08g21430D	SANT/Myb domain
	C08	25243813	4.31-10.90	1.53-5.04	2,3,4	E3	BnaC08g21500D	Cyclin, C-terminal domain
							BnaC08g22620D	Auxin responsive SAUR
**SI**	A01	21122950	3.67–7.27	0.51–1.51	2,4,5	E1	BnaA01g31080D	SANT/Myb domain
	A01	21380473	3.07–7.82	0.46–3.18	3,4,5	E3	BnaA01g31290D	AP2/ERF domain
							BnaA01g31770D	F-box domain, cyclin-like
	C02	15059835	4.29–17.47	1.57–6.12	2,4,5	E3	BnaC02g18860D	CWC16 protein
	C02	16019162	3.41–8.08	1.92–3.15	3,4,5	E2	BnaC02g19100D	SBP-box
							BnaC02g19360D	MADS-box
	C03	21828785	3.50–5.00	1.03–2.84	2,3,4	E2	BnaC03g36030D	Armadillo-like helical
	C03	22767136	3.07–6.32	0.27–2.16	1,2,3,4,5	E3	BnaC03g36600D	No apical meristem
							BnaC03g36740D	DNA topoisomerase
**SN**	A02	13065894	4.29–5.52	1.00–1.89	2,4,5	E1	BnaA02g20660D	Myb/SANT-like domain
	A02	13595586	5.59–8.57	2.44–2.85	2,3,5	E2	BnaA02g20930D	F-box domain, cyclin-like
							BnaA02g21200D	MADS-box
	C06	13960751	3.85–4.74	1.64–2.59	2,4,5	E2	BnaC06g11920D	Protein kinase
	C06	14647381	4.26–7.57	1.76–3.24	2,3,4,5	E3	BnaC06g12120D	Sucrose synthase
	C07	3019188	4.02–6.54	0.92–2.52	2,3,4	E3	BnaC07g02320D	F-box domain, cyclin-like
	C07	3183504	4.18–6.15	1.32–2.43	1,2,4,5	E2	BnaC07g51040D	GH3
							BnaC07g51180D	Aminotransferase
	C09	11236617	6.51–9.86	3.23–3.96	1,2,3	E2,E3	BnaC09g14450D	PI4P5K
	C09	16839183	4.55–9.55	128–4.06	2,4,5	E2,E3	BnaC09g14650D	Dynamin central domain
	C09	20774003	3.19–5.58	0.88–2.36	2,4,5	E2,E3	BnaC09g14760D	SANT/Myb domain
							BnaC09g19820D	JmjC domain
**SP**	C03	30605297	5.00–6.14	0.98–2.41	2,3,5	E3	BnaC03g45540D	AGP9
	C03	31339715	4.03–8.30	2.14–4.80	1,2,3,4,5	E2	BnaC03g45610D	GASA7
							BnaC03g46070D	F-box, cyclin-like
							BnaC03g46450D	SAUR protein
	C09	24974617	3.45–6.37	1.45–3.28	2,3,4	E2	BnaC09g25390D	LTPG4
	C09	25836173	3.13–11.90	3.46–5.67	2,3,4,5	E1	BnaC09g25980D	SWEET sugar transporter
							BnaC09g26050D	Peptidase C48, SUMO
							BnaC09g26100D	Protein phosphatase 2C
**SW**	A02	7610485	7.18–21.14	1.77–8.32	1,2,3,4,5	E1,E3	BnaA02g13530D	SAM methyltransferase
							BnaA02g13870D	Zinc finger, C2H2
							BnaA02g13950D	AP2/ERF domain
	A06	15184188	6.07–14.12	1.68–4.73	1,2,3	E1,E3	BnaA06g21690D	Protein kinase
							BnaA06g21720D	DUF296
							BnaA06g21890D	Zinc finger, RING-CH-type
	A09	27915980	3.85–11.85	2.11–3.22	3,4,5	E3	BnaA09g39450D	Cytochrome b561
	A09	28182807	5.35–9.51	2.44–3.16	1,2,4	E2	BnaA09g39480D	No apical meristem (NAM)
	A09	28130192	4.00–26.19	3.21–7.50	2,3,4,5	E1,E3	BnaA09g39620D	HAD-hydrolase
							BnaA09g39680D	AUX/IAA protein
							BnaA09g39790D	Helicase, C-terminal
							BnaA09g39840D	PMR5 N-terminal domain
							BnaA09g39950D	Lipase, class 3
**YP**	A07	21399751	5.25–8.52	0.76–4.22	2,3,5	E3	BnaA07g29760D	ABC-2 type transporter
	A07	21594406	4.46–8.12	1.29–1.91	1,2,3,4	E2	BnaA07g30510D	Toll/interleukin-1 receptor
							BnaA07g30950D	Auxin efflux carrier
	A10	8882087	3.20–4.74	0.34–4.04	1,2,4,6	E3	BnaA10g10240D	No apical meristem (NAM)
	A10	9777221	3.93–6.97	2.08–3.50	1,3,4	E1	BnaA10g10870D	Acyl-CoA N-acyltransferase
							BnaA10g11980D	SANT/Myb domain
	C03	44895348	3.45–11.09	1.66–5.47	1,2,3,4	E3	BnaC03g55830D	E3 UFM1-protein ligase 1
	C03	45022247	3.26–4.14	1.06–2.63	2,3,4	E1	BnaC03g55940D	Protein kinase

Chr, Chromosome; Env, Environments; PH, Plant height; FT, Flowering time; BN, Primary branch number; SI, Silique number of main inflorescence; SN, Silique number per plant; SP, Seed per pod; SW, 1000-seed weight, YP, Yield per plant. E1, YL13; E2, NC14; E3, WH16.

Forty QTNs shared by ML-GWAS and SL-GWAS within the 1 Mb region were found to associate with the eight yield traits, with the highest number of QTNs associated with SW (10) and the lowest with SI (2) (**Supplementary Table S4**) (Hu et al., 2022). Among the overlapped QTNs with SL-GWAS, 10 QTNs including A09: 28130192 and A09:2818207 associated with SW were simultaneously detected, and some of them were found in at least two environments, suggesting that these QTNs were more stable and reliable (**Table 2** and **Supplementary Table S4**). Four QTNs were detected in all five ML-GWAS and SL-GWAS, and two of them, both associated with SW, were found repeatedly in two environments, explaining 4.10 ~ 22.24, 5.32 ~ 10.60 of the phenotypic variation, respectively (**Supplementary Table S4**). Notably, we detected two significant signal loci, A09:28182807 and C09:25836173, in three environments simultaneously, associated with SW and SP, respectively. The two loci have high LOD values and may be significant trait-associated QTNs with breeding potential (**Supplementary Figure S3**). Thus, a total of 24 and 15 stable significantly associated QTNs were detected by ML-GWAS for SW and SP, explaining 0.24 ~ 8.32 and 0.00 ~ 6.74 of phenotypic variation, respectively (**Supplementary Table S5**).

**Table 2:** Significant 1000-seed weight (SW) associated QTNs and candidate genes detected in at least two environments by multi loci-GWAS.

Chr.	Position	Allele	LOD	*R* ^2^(%)	―log10(*P*)	Environment	Methods	MLM (Position)
A02	7610485	T/A	3.39–21.14	4.10–22.24	0.83–8.32	E1,E3	1,2,3,4,5	7600283~7620485
A02	9978238	A/G	3.34–10.95	4.05–11.91	0.89–1.86	E1,E2,E3	1,3,5	9972386~12256474
A04	6184539	T/G	4.18–8.10	4.67–9.00	1.95–2.93	E1,E2,E3	1,2,3	
A05	11305754	C/T	3.30–4.6	4.02–5.43	0.88–2.24	E1,E3	1,2,3,5	
A06	10063511	A/T	7.40–10.03	8.27–10.97	2.43–5.21	E2,E3	1,2,5	
A06	15184188	C/T	4.04–14.12	4.79–15.13	0.82–4.73	E1,E3	1,2,3	
A09	4168712	A/G	3.08–0.34	3.79–11.41	0.99–4.50	E2,E3	1,2,3,4	
A09	28130192	C/T	4.00–26.19	4.75–27.33	1.17–7.50	E1,E2	2,3,4,5	28085608~28158503
A09	28182807	A/G	5.35–17.43	6.16–18.48	2.44–4.26	E1,E2	1,2,4	28171742~28199140
A10	15198168	T/C	3.63–12.37	4.14–13.35	1.11–4.66	E1,E3	2,4,5,6	
C01	11143808	A/C	3.63–7.30	4.36–8.18	1.62–1.92	E1,E2	1,3,5	11143808~15054178
C03	25856914	G/T	4.54–9.67	5.32–10.60	1.94–4.54	E1,E3	1,2,3,4,5	25846914~25866982
C03	33059809	G/A	3.87–8.18	4.62–9.08	0.24–0.65	E1,E3	1,2,3	
C03	38304116	T/G	3.05–7.82	3.05–7.82	1.58–3.51	E1,E2	2,4,5	
C05	6286803	T/C	4.27–5.69	5.03–6.51	1.75–3.62	E1,E2,E3	2,4,5	
C05	30666036	T/A	3.79–6.92	4.53–7.78	1.63–3.23	E1,E3	2,4,5	29675390~30666036
C07	18309538	C/T	3.37–6.54	4.00–7.75	1.04–3.07	E1,E2,E3	2,3,4,5	18306538~22307432
C07	37609075	C/A	5.93–8.90	6.77–9.81	0.88–6.79	E2,E3	1,2,4,5	
C07	38545053	A/G	3.23–10.84	3.23–10.84	0.87–3.68	E1,E3	1,2,3,4	
C08	18157206	C/T	4.50–8.45	5.27–9.35	1.94–5.61	E2,E3	1,2,4	
C08	37165475	A/G	5.33–6.35	6.14–7.19	1.37–4.21	E2,E3	1,2,3,4	31608617~37165765

The six ML-GWAS methods: 1, mrMLM; 2, FASTmrMLM; 3, FASTmrEMMA; 4, pLARmEB; 5, pKWmEB; 6, ISIS EM-BLASSO. E1, YL13; E2, NC14; E3, WH16.

Since SW is the important component of yield traits, and is relatively stable in different environments in this study, we further analyzed the QTNs associated with SW. A total of 21 significant QTNs were identified simultaneously in at least 2 environments as well as in three models (**Table 2**). And nine of the 21 QTNs were also detected by SL-GWAS with MLM model (**Table 2**), indicating the reliability of ML-GWAS (Hu et al., 2022). A roughly similar number of QTNs was found in each environment, and the results were also similar for each model, except for pLARmEB, which identified only one. These QTNs were randomly distributed on different chromosomes and their LOD scores ranged from 3.05 ~ 26.19, explaining 3.05 ~ 27.33 of the phenotypic variation (**Table 2**).

### Allelic effects of stable QTNs for yield traits

**Figure 3:**
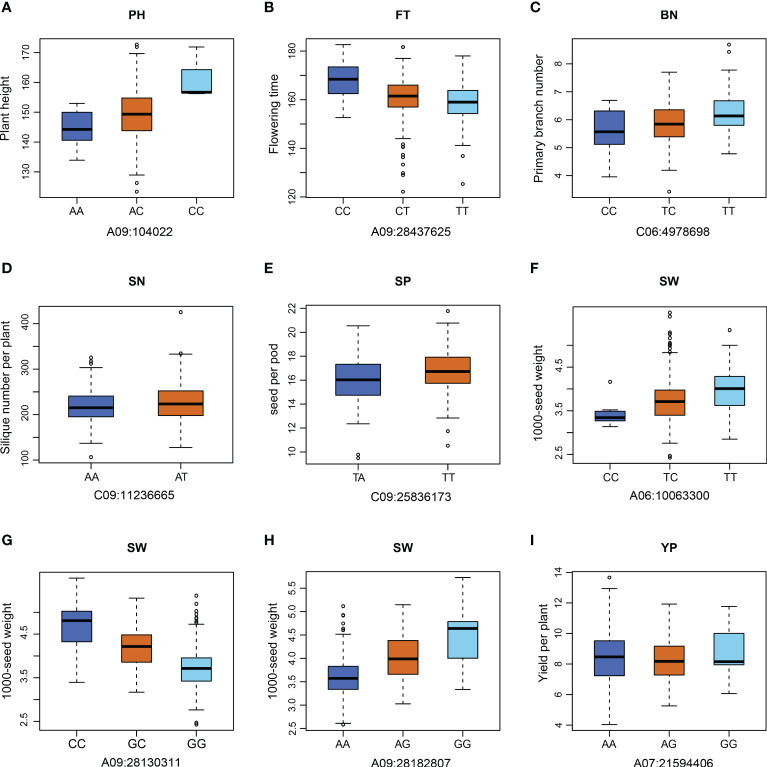
Phenotypic differences between two or three genotypes for each of the nine QTNs. **(A-I)** Phenotypic variations at different alleles of nine QTNs for the yield traits. PH, plant height; FT, flowering time; BN, primary branch number; SN, Silique number per plant; SP, seed per pod; SW, 1000-seed weight, YP, yield per plant.

**Figure 4:**
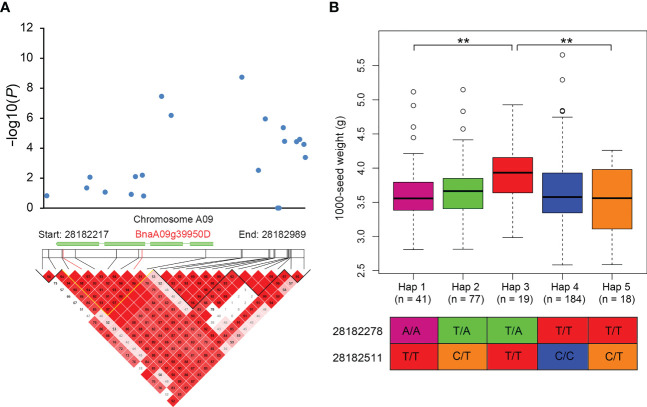
The QTN detected in at least two environments on chromosome A09 for 1000-seed weight (SW) with LD heatmap surrounding the QTN. **(A)** Manhattan plot of the A09 chromosomal region around the candidate gene *BnaA09g39950D* and LD heatmap with a peak SNP (A09: 28182807). **(B)** Haplotype analysis of *BnaA09g39950D*. Box plots show the distribution of each haplotype group, n denotes the number of genotypes belonging to each haplotype group and the genotypes less than ten are not shown. ** Significant differences between the haplotypes were evaluated by two-tailed *t* test (*P* < 0.01).

To identify the favorable alleles of QTNs for the eight yield traits in rapeseed, we analyzed the significant phenotypic differences between the elite and alternative alleles of 21 QTNs (**Figure 3** and **Supplementary Figure S4**). We divided the population into two or three groups according to their allele types and compared the phenotypic values among the different groups. Generally, population with elite alleles had significantly larger values than those with unfavorable alleles. For example, accessions with the TT allele of C09:25836173 show more SP compared to those with the AA variant, indicating TT could be considered an elite allele. We focused on A09:28182807, a significant locus associated with SW, and the average SW values of GG individuals of A09:28182807 was significantly higher than that of AA and AG individuals. We further analyzed the haplotype blocks in the 772 bp region around the peak locus A09:28182807 and identified a strongly linked block (**Figure 4A**). Based on the genotypes of this block, the association population of rapeseed germplasms was divided into five major haplotype groups with two nonsynonymous SNPs (**Figure 4B**). Haplotype Hap 3 (n =19) had relatively large SW values and showed significant differences from Hap 1 (n = 41) and Hap 5 (n = 18) (**Figure 4B**). These results suggest that locus A09:28182807 significantly associated with SW and that the haplotype Hap 3 of *BnaA09g39950D* may increase seed weight.

Twelve other QTNs associated with the seven yield-related traits PH, FT, SI, BN, SN, SW and YP were further analyzed, indicating some elite alleles improve the yield traits (**Supplementary Figure S4**). The favorable alleles obviously affected FT with the population with the AA elite allele at A03:22616177 having a mean FT of around 170 days, compared to around 160 days for the CC allele group (**Supplementary Figure S4**). These findings suggested that the accessions with elite alleles have clearly higher phenotypic values for yield-related traits compared to those with unfavorable allelic variations. Nineteen important QTNs shared by multiple environments and multiple methods regulate five important yield-related traits, including SW, FT, BN, SI, and SN. And these QTNs were used to assess the utilization of favorable alleles in 20 elite accessions during rapeseed breeding (**Supplementary Figure S5**). Among these elite accessions, the number of superior alleles in the QTNs ranged from 5 (26.3%, Shengliqinggeng) to 12 (63.2%, Zhongshuang 11), of which seven accessions had more than 10 (52.6%) superior alleles. In addition, we found that some superior alleles of the QTN loci were prevalent in these accessions, for example, the superior alleles of C09:11236665 and C09:11236689 were in almost all the accessions (19/20). These results suggest that some common elite alleles may have a particularly large impact on yield.

### Identification of candidate genes based on stable QTNs and transcriptome analysis

Considering the LD decay distance of the rapeseed population, the regions within 200-kb on either side of the stable QTNs based on ML-GWAS were used to identify the candidate genes. Thus, 4796 genes were mined surrounding the 75 stable QTNs identified by ML-GWAS in different environments (**Table 1** and **Supplementary Tables S4, S5**). There are many candidate genes involved in plant growth and development, such as *BnaA07g30950D* (Auxin efflux carrier), which is involved in maintaining the embryonic hormone gradient, and also involved in shoot and root development. The genes *BnaA07g30180D* (SAUR protein), *BnaA09g39680D* (AUX/IAA protein), *BnaA09g40340D* (F-box domain, cyclin-like) and *BnaC03g46450D* (SAUR protein) have all been implicated in the regulation of plant growth (**Table 1**). Furthermore, 1807 genes were found in the QTNs of SW and 942 genes were discovered in SP (**Supplementary Figure S6** and **Table S5**). Haplotype analysis of the candidate genes *BnaA06g17710D*, *BnaA09g39450D* and *BnaA09g39950D* for SW showed that the different haplotypes had significant phenotypic difference (**Supplementary Figure S7**). In the significant QTN cluster (A09: 27915980 ~ 28130192 ~ 28182807) for SW, which was also detected by SL-GWAS, three candidate genes *BnaA09g39450D* (Cytochrome b561), *BnaA09g39790D* (RNA helicase), and *BnaA09g39950D* (Lipase) were identified to associate with SW (**Figures 3**, **4** and **Supplementary Table S5**).

**Figure 5:**
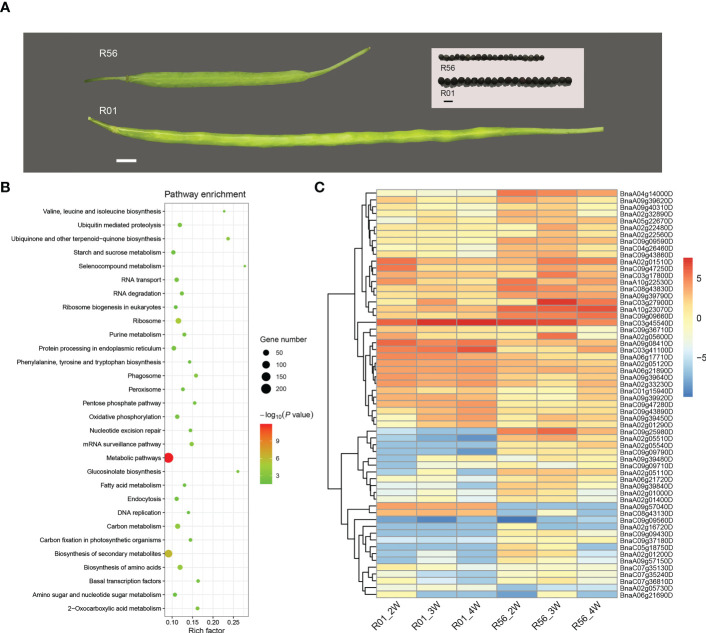
Transcriptome analysis of two rapeseed accessions with extremely seed size/seed weight. **(A)** Seeds and siliques of R01 (large seed) and R56 (small seed) showed considerable variations in seed weight. **(B)** The top 30 significantly enriched KEGG pathways of differentially expressed genes (DEGs) between R01 and R56 at three stages. **(C)** Heatmap of the expression patterns of the 60 genes in developing seeds of two rapeseed cultivars at three stages. The red indicates high expression, and the blue shows low expression. R01_2W, R01_3W, R01_4W, R56_2W, R56_3W and R56_4W represent the sampling time of R01 and R56 are two weeks after pollination, three weeks after pollination, and four weeks after pollination, respectively.

To further determine the candidate genes associated with the two important traits SW and SP, we analyzed transcriptomic data from two accessions with extremely difference of seed size, R01 and R56. The accession R01 with larger seeds than that of R56, and has significant different seed number per pod (R01, n = 14.74 *vs* R56, n = 20.56) (**Figure 5A**) (Hu et al., 2022). We found a total of 2572 differentially expressed genes (DEGs) among three different stages in these two accessions (**Supplementary Figure S6** and **Table S6**). Searching for commonly identified genes in the DEGs and the candidate genes for ML-GWAS results, we identified 79 reliable candidates with 50 and 29 of which regulate SW and SP, respectively (**Supplementary Figures S6B, C** and **Tables S7**, **S8**). GO enrichment analysis revealed that DEGs were enriched to fruit development terms (GO: 0010154) in biological processes (GO: 0008150) and identified 76 associated genes (**Supplementary Figure S6**). KEGG analysis indicated that a large number of DEGs were involved in the metabolic pathways and biosynthesis of secondary metabolism (**Figure 5B**). Moreover, the gene annotation of DEGs demonstrates considerable genes related to plant hormones, especially auxin, as well as an abundance of transcription factors (TFs).

### Analysis of candidate genes expression patterns

Expression pattern analysis was performed for 60 genes detected in both DEGs and ML-GWAS for SW and SP (**Figure 5C**). We identified a number of genes that were highly significantly differentially expressed in the two cultivars R01 and R56 (**Figure 5C**). *BnaC09g25980D* (*SWEET7*) is a candidate gene for SP, which is extremely highly expressed in R56 plants and lowly expressed in R01 (**Figure 5C**). In addition, *BnaA02g05510D*, *BnaA02g05540D*, and *BnaC09g09790D* also had similar expression patterns (**Figure 5C**). However, *BnaA09g57040D* and *BnaC08g43130D* had opposite expression profiles, both of which were highly up-regulated in R01 and lowly expressed in R56. There are also many genes to be up-regulated in both accessions, but they are more remarkable in R01 than in R56. The SW-related candidate gene *BnaA09g39450D* (Cytochrome b561) was more highly expressed in R01, and expression levels increased progressively with developmental time (**Figure 5C**).

We next investigated the expression profiles of genes related to different phytohormones, such as auxin, jasmonic acid (JA) and gibberellin (GA), cyclin-related genes, and TFs in the 2572 DEGs (**Supplementary Figures S8A–E**). Transcripts of seven auxin-related genes were up-regulated in R56 compared to R01, for example, *BnaA05g00250D*, *BnaC04g00140D* and *BnaC09g00360D* had higher expression levels in R56 (**Supplementary Figure S8A**). All three genes related to JA were extremely up-regulated in R56, but *BnaA02g05120D* was expressed at a higher level in R01 (**Supplementary Figure S8B**). Three genes related to, GAs especially *BnaC03g11560D* were also up-regulated in R56, but two cyclin genes, *BnaC01g38940D,* and *BnaC02g02720D*, were significantly higher expressed in R01 than in R56 (**Supplementary Figures S8C, D**). In addition, 36 TFs in 2572 DEGs were showed differentially expressed in R01 and R56 (**Supplementary Figure S8E**).

### Validation of DEGs by qRT-PCRs

**Figure 6:**
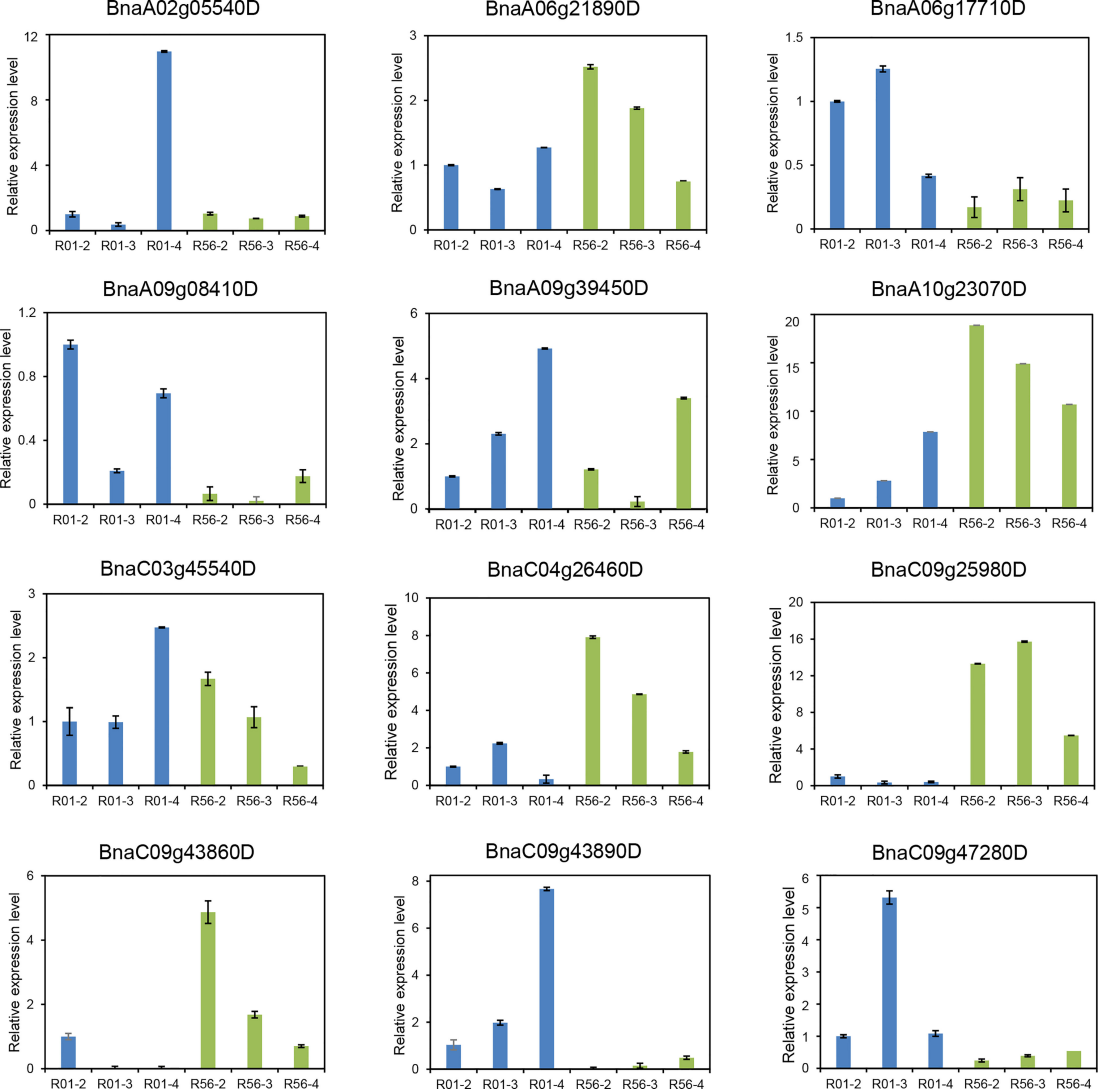
Quantitative RT-PCR (qRT-PCR) validation of differentially expressed genes (DEGs) for 1000-seed-weight (SW) between R01 and R56 from RNA-seq data. The transcript abundances were calculated from three replicates with *BnACTIN7* (*BnaA03g55890D*) as internal control. Data are shown as means ± SE. R01-2, R01-3, R01-4, R56-2, R56-3 and R56-4 represent the sampling time of R01 and R56 are two weeks after pollination, three weeks after pollination, and four weeks after pollination, respectively.

Twelve candidate genes were selected for qRT-PCR analysis based on the combined results of ML-GWAS and RNA-seq (**Figure 6**). Overall, the expression levels of these genes were diverse in the two accessions and varied across developmental periods. The expression profile of the candidate gene *BnaC09g25980D* for SP was consistent with RNA-seq, with both showing high expression levels of in R56 and lowly expression in R01. The qRT-PCR validation of the candidate gene *BnaA09g39450D* at the significant signal locus A09:28182807 for SW showed that it was expressed in both accessions, but transcripts were more abundant in R01 and increased progressively over time. Interestingly, the genes *BnaC09g43860D* and *BnaC04g26460D* were lowly expressed in R01 and expressed at high levels in R56, but gradually decreased in R56 over time. There are three genes, such as *BnaA06g21890D*, *BnaC03g45540D* and *BnaA10g23070D*, which are expressed in both accessions and their expression levels increase progressively with development in R01 but decrease progressively in R56. For gene *BnaA02g05540D*, it was extremely lowly expressed at different developmental periods in both R01 and R56, except for R01 at 4WAP. In addition, we found that most of these genes were significantly differentially expressed in both accessions, such as *BnaA06g17710D*, *BnaA09g08410D*, *BnaC09g43890D,* and *BnaC09g47280D*.

## Discussion

Seed yield is an important and complex quantitative trait in rapeseed. And developing high yield rapeseed varieties is the goal for breeders in many cases. Previous studies did not comprehensive analysis of the seed yield and yield-related traits in different environments at the same time using whole genome resequencing. In the present study, we not only examined the relationship among eight yield traits, but also detected novel QTNs for the yield traits in three environments. These findings would be helpful for breeders to develop rapeseed varieties by congregating superior alleles.

In rapeseed, lots of complex traits have been dissected *via* the GLM or MLM based on the single-locus using arrays or resequencing data (Lu et al., 2019; Song et al., 2020; Wang et al., 2021a; Hu et al., 2022). However, using one model for GWAS has limitations and may miss the small-effect loci (Ma et al., 2018). In this study, we use multi-locus methodologies, including mrMLM, FASTmrEMMA, pLARmEB and so on, to detect novel and more loci for seed yield and yield-related traits in rapeseed. Using ML-GWAS, a total of 908 QTNs were identified by at least two of six ML-GWAS methods and 596 QTNs of them were obtained for integrating multiple approaches (**Figure 2** and **Supplementary Table S2**). And 40 loci were the same and near the loci which have been detected in rapeseed using SL-GWAS with MLM model as previous study (Hu et al., 2022). Furthermore, 75 of new QTNs have been identified in different environments and at least two models (**Supplementary Table S3**). Meanwhile, each method successfully detected some loci which other methods were not identified, indicating that it is worth using various methods for GWAS analysis (Liu et al., 2020). The stable QTNs identified in two or more environments or at least two methods increased the reliability of these loci. Using these methods, many novel loci were discovered for the seed yield and yield related traits of rapeseed (**Supplementary Tables S2**-**S4**). Thus, these ML-GWAS approaches would be effective alternative methods to dissect the genetic architecture for agronomic traits. In addition, integration of GWAS and transcriptomic analysis could reliably identify candidate genes for seed yield in rapeseed (Lu et al., 2017; Zhang et al., 2022a).

Seed size, an important agronomic trait determining crop yield, affects the SW and SP in rapeseed. In the significant QTN cluster on chromosome A09 for SW, three candidate genes *BnaA09g39450D* (Cytochrome b561), *BnaA09g39790D* (RNA helicase), and *BnaA09g39950D* (Lipase) were identified to affect seed size (**Figures 3**, **4** and **Supplementary Table S4**). Cytochrome b561, which plays an important role in plant growth and development has been also identified the QTL *qGY8.1* for yield in rice (Balakrishnan et al., 2020). In our study, the expression level of *BnaA09g39450D* was validated to be higher in R01 than that in R56 (**Figure 6**). In *Arabidopsis*, RNA helicase has been reported to participate in coordination between cell cycle progression and cell size, which is required for ovule development and involved in seed size regulation (Yoine et al., 2006; Bush et al., 2015). Lipase is reported to involve in seed oil production in many plants (Eastmond, 2006; Wang et al., 2021b). In most cases, the seed oil content positively correlated with seed size in *B. napus*. For example, six nonspecific phospholipase C (NPC) genes have been identified to associate with SW or YP and the favorable haplotype of *BnNPC6.C01* could increase seed oil content and seed yield (Cai et al., 2020). Patatin-related phospholipase pPLAIIIδ has been reported to affect organ size (silique) in *Arabidopsis* and *B. napus* (Dong et al., 2014). Therefore, these three candidate genes might be involved in the regulation of seed weight and seed yield in rapeseed. Seed size is also determined by the carbohydrate *via* the phloem to developing seeds during seed filling (Sosso et al., 2015). In soybean, both GmSWEET10a and GmSWEET10b were shown to transport sucrose and hexose, contributing to sugar allocation, which consequently simultaneous increases oil content and seed size in soybean (Wang et al., 2020). In our study, the candidate gene *BnaC09g25980D* (*SWEET7*) was validated to be highly expressed in R56 (**Figure 6**), indicating that *SWEET7* might regulate the seed size in rapeseed.

Many plant hormones have been reported to be involved in the regulation of seed size, including auxin pathway, GA signaling and brassinosteroid (BR) signaling (Li et al., 2019c). In this study, using the extremely difference of seed size accessions R01 and R56 with significantly different seed number per pod, 2572 DEGs were identified by RNA-seq (**Figure 5** and **Supplementary Figure S6**). And we identified 58 DEGs related to phytohormones, cell cycle and TFs including NAC, TCP, MYB and so on (**Supplementary Figure S8** and **Table S5**). Auxin is known to regulate plant growth and development *via* cell division and cell elongation (Hu et al., 2021b). In previous studies, auxin signaling genes *ARF18* and *BnaA3.IAA7* have been reported to regulate seed weight and yield in *B. napus* (Liu et al., 2015; Li et al., 2019b). In our study, several candidate genes and ten DEGs were found to involve in auxin signaling pathway (**Table 1**, **Supplementary Figure S8** and **Table S6**). The GASA family in *Arabidopsis* is regulated by GA, with the GASA4 mutant having smaller seeds than the wild type and increased grain weight after overexpression (Roxrud et al., 2007). A number of GA-related candidate genes, including *GASA10* (*BnaC03g11560D*) was highly expressed in R56, which may affect the seed size (**Supplementary Figure 8C**). Hu et al. (2021c) revealed that JA signaling represses seed size and negatively regulates cell proliferation of integument during seed development. The JA signaling repressor *jaz6* mutants in *Arabidopsis* exhibited small seed size, and overexpression of *BnC08.JAZ1-1* in *Arabidopsis* resulted in enhanced seed weight (Hu et al., 2021c; Wang et al., 2022). In this study, *JAZ12* (*BnaA02g05120D*) and *JAZ 9* (*BnaA07g28810D*) were up-regulated in R01, while another candidate gene *JAZ10* (*BnaC09g43860D*) was highly expressed in R56 (**Figure 6**, **Supplementary Figure 8B** and **Table S6**). These results suggested that auxin, GA, and JA signaling genes were involved in the control of seed size and seed number per pod in rapeseed. Furthermore, we observed that several cell cycle genes, including *BnaC04g26460D* (*CDKB1;1*) were up-regulated in R56 (**Figure 6** and **Supplementary Figure S8**), indicating these DEGs play important roles in cell division and seed size (Qi et al., 2012; Hu et al., 2021c). In addition, one of the candidate gene *BnaC09g43890D* (*NAC083*) was validated highly expressed in R01 (**Figure 6**). In rice, three NAC genes *NAC020*, *NAC026* and *NAC023* have been reported to associate with seed size/weight (Mathew et al., 2016). And *VvNAC26* was also demonstrated to regulate the seed size by interacting with VvMADS9 in grapevine (Zhang et al., 2021). Therefore, further functional studies of these genes associated with yield traits will help to elucidate the mechanism of high yield and apply to develop high-yielding rapeseed varieties.

## Conclusion

In this study, a total of 908 QTNs were detected for eight yield traits using two or more ML-GWAS methods. Of them, 75 stable QTNs (or QTN clusters) controlling yield traits were obtained with a significant QTN cluster on chromosome A09 for SW, which was also identified by SL-GWAS. Twenty elite rapeseed accessions had a diverse distribution of superior alleles, and the high-yielding accessions contained more superior alleles. Integrated ML-GWAS with transcriptome analysis, 79 candidate genes were found to associate with SW or SP. Some genes related to plant hormones such as auxin, JA, and GA, were involved in the regulation of rapeseed yield. Thus, many robust QTLs with candidate genes were identified to regulate seed size and yield traits in rapeseed. This study made a beneficial attempt *via* a combinatory approach of ML-GWAS methods to facilitate the detection of yield-related QTNs in rapeseed. These findings will provide valuable information for understanding the mechanism underlying seed yield and yield-related traits and accelerate the crop improvement of rapeseed.

## Data availability statement

The datasets presented in this study can be found in online repositories. The names of the repository/repositories and accession number(s) can be found in the article/**Supplementary Material**.

## Author contributions

JH and SL designed and supervised the research. SL, HZ, JH, YL and CD performed multi-locus GWAS and bioinformatics analysis. CZ, RG, RZ, JD and JH performed the experiments and data analysis. CZ, RZ, JH and HZ wrote and revised the manuscript. All authors contributed to the article and approved the submitted version.
