# Supplementary material for: Integrated multi-locus genome-wide association studies and transcriptome analysis for seed yield and yield-related traits in Brassica napus
Source: Front Plant Sci. 2023 Apr 14;14:1153000. doi: 10.3389/fpls.2023.1153000 (PMC10140536; doi:10.3389/fpls.2023.1153000)
Supplement: Supplementary file 2 [file DataSheet_1.pdf]

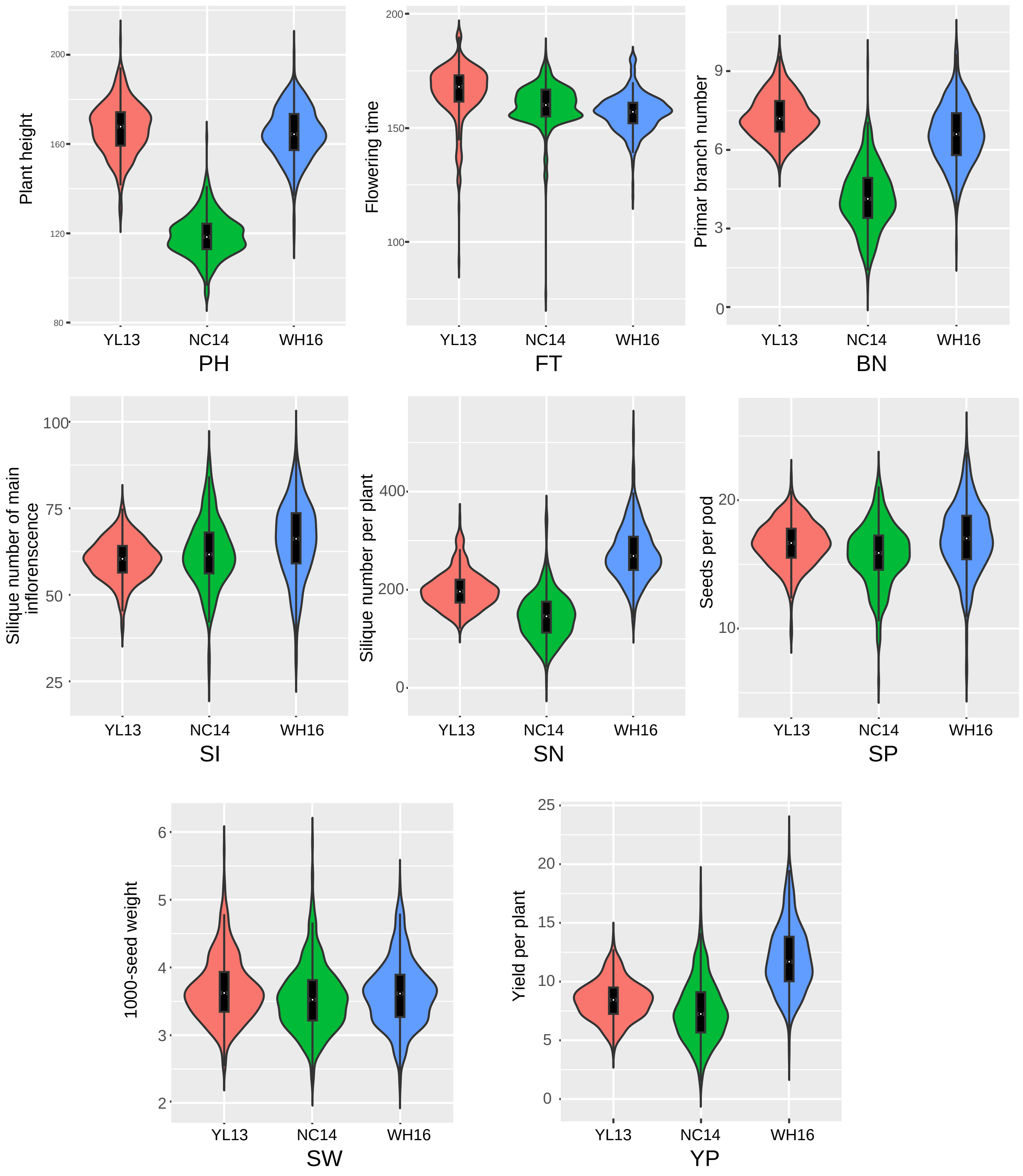

Figure S1 Violin plots show the distributions of values for eight yield-related traits (PH: Plant height; FT: Flowering time; BN: Branch number; SI: Silique number of main inflorenscence; SN: Silique number per pods; SP: Seeds per pod; SW: 1000-seed weight; YP: Yield per plant) of rapeseed in three environments (Yangluo 2013,YL13; Nanchang 2014, NC14; Wuhan 2016, WH16).

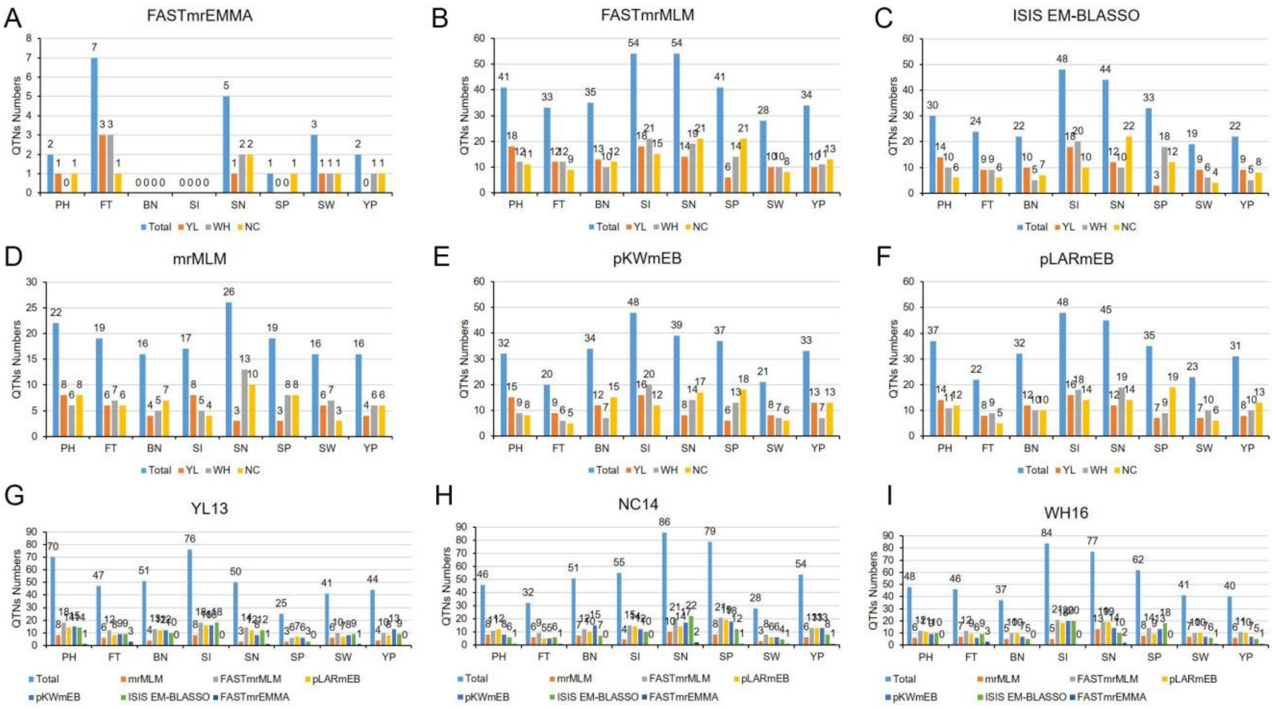

Figure S2 Number of detected QTNs for the eight traits across three environments in six methods. The traits include PH (plant height), FT (flowering time), BN (branch number), SI (silique number of panicle), SN (silique number per plant), SP (seed per pod), SW (1000-seed weight), and YP (yield per plant). YL, WH, and NC indicate the population planted in Yangluo (2013), Nanchang (2014), and Wuhan (2016), respectively. The approaches include (A) FASTmrEMMA, (B) FASTmrMLM, (C) ISIS EM-BLASSO, (D) mrMLM, (E) pKWmEB, (F) pLARmEB. (G-I) Comparison of the number of detected QTNs from the six methods. The environments include Yangluo (YL2013) (G), Nanchang (NC2014) (H), and Wuhan (WH2016) (I). Total: represents the total QTNs number for each trait.

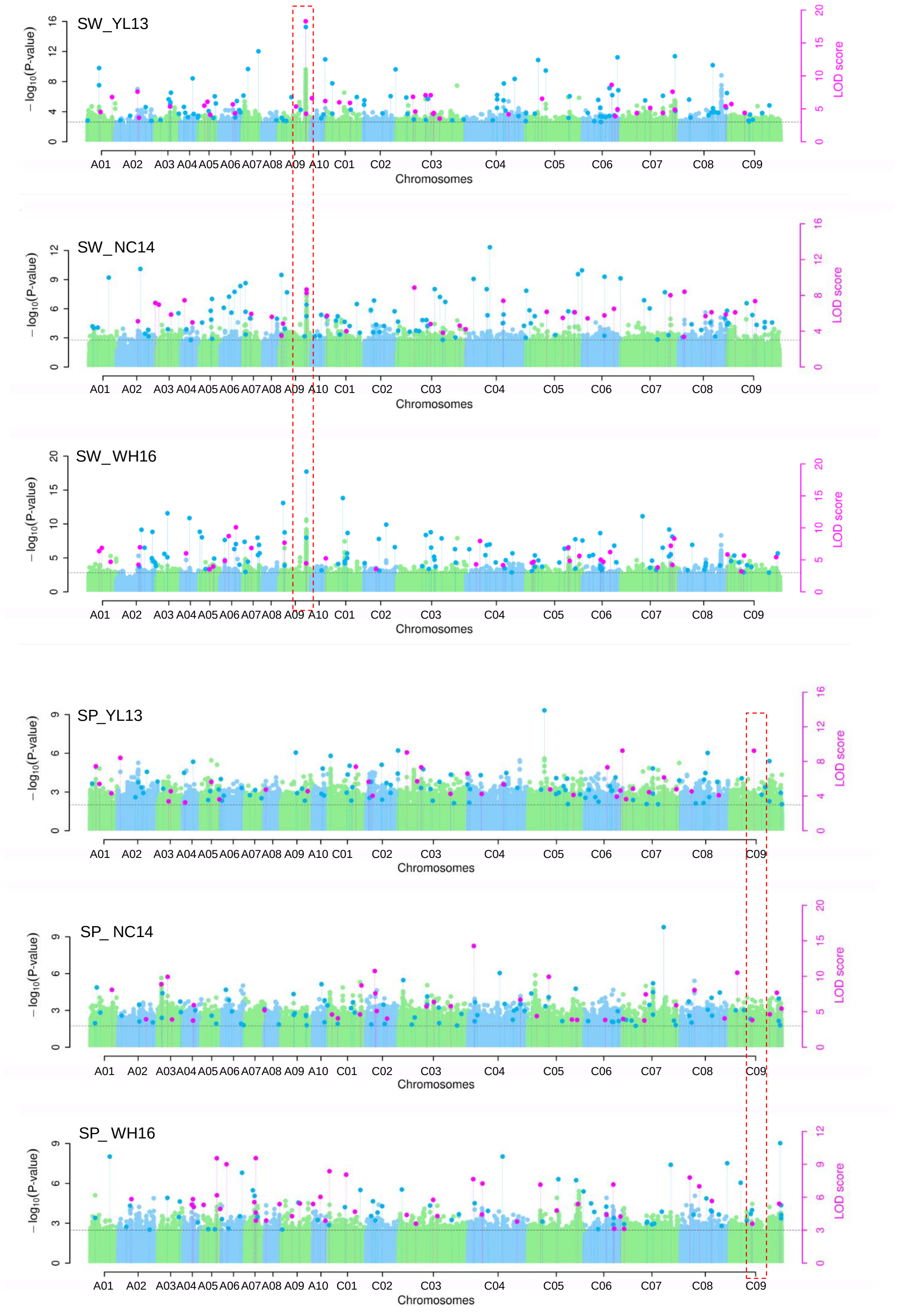

Figure S3 Manhattan plots of ML-GWAS for 1000-seed weight (SW) and seed per pod (SP) in three three environments. The dashed lines indicate the threshold values for SW and SP. Different moldes: 1, FASTmrEMMA; 2, FASTmrMLM; 3, ISIS EM-BLASSO; 4, mrMLM; 5, pKWmEB; 6, pLARmEB.

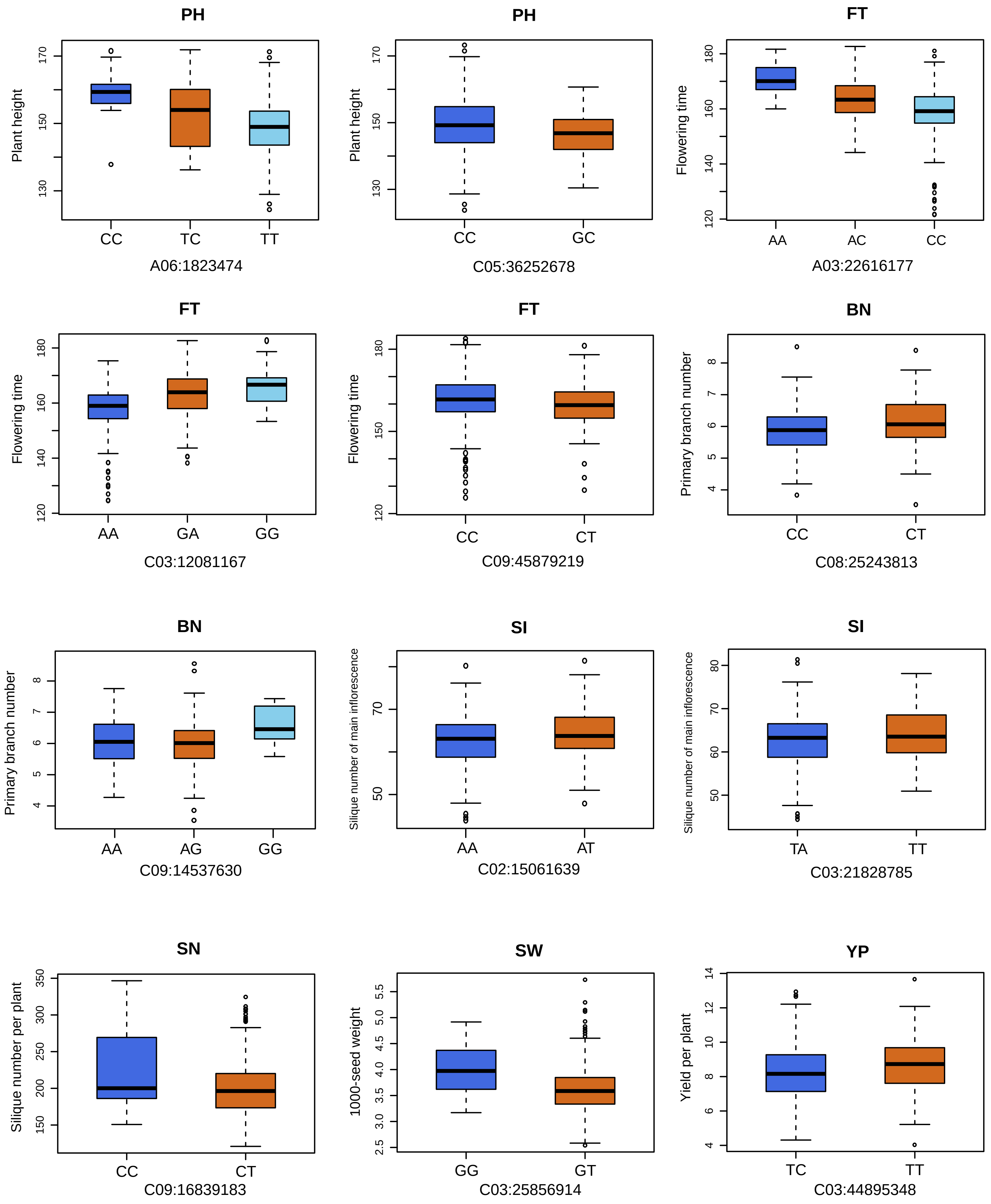

Figure S4 Phenotypic differences between two or three genotypes for each of the twelve QTNs.

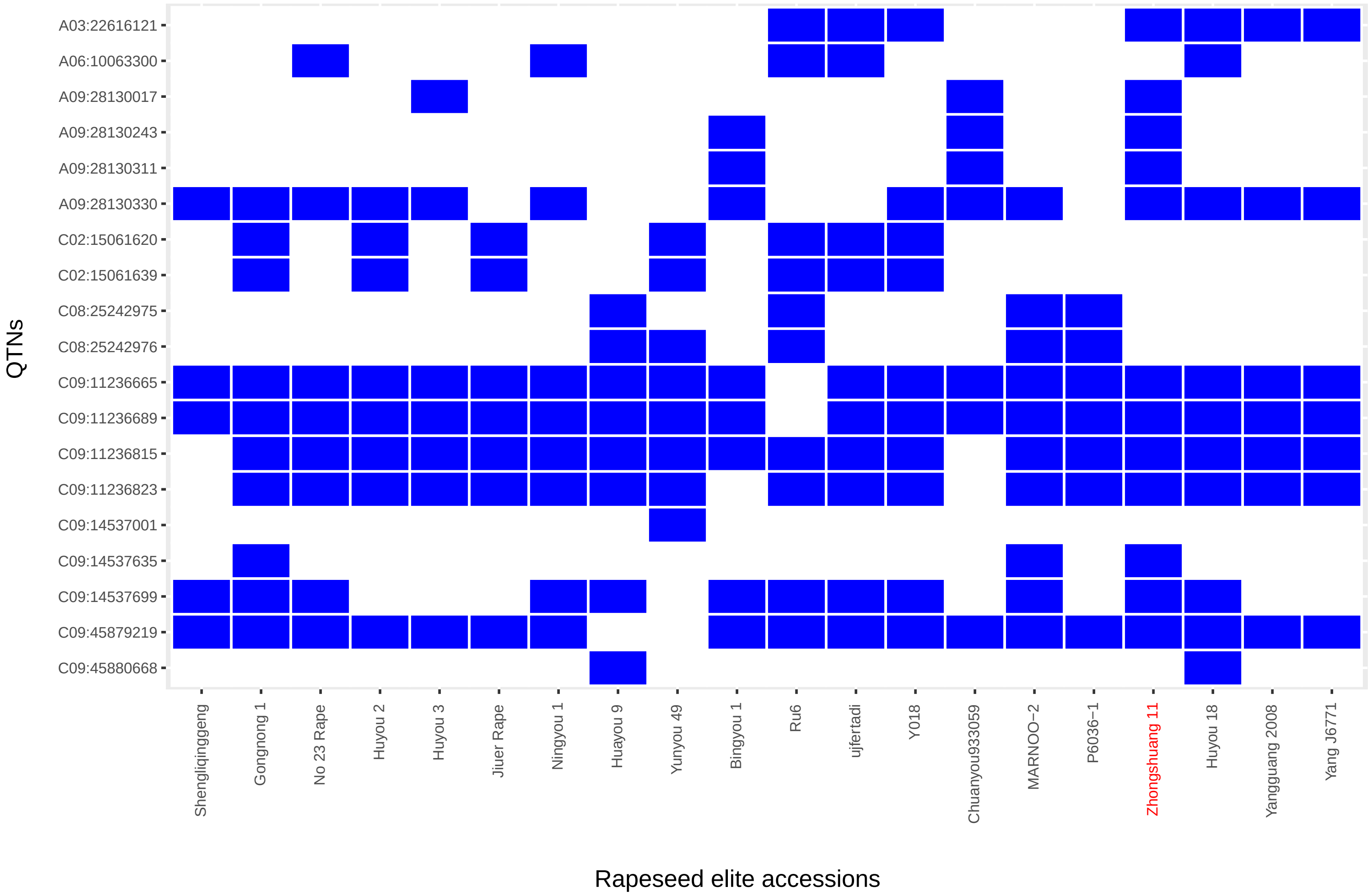

Figure S5 The superior alleles distributions in the 20 rapeseed accessions. Blue and white colours represent superior and inferior alleles, respectively.

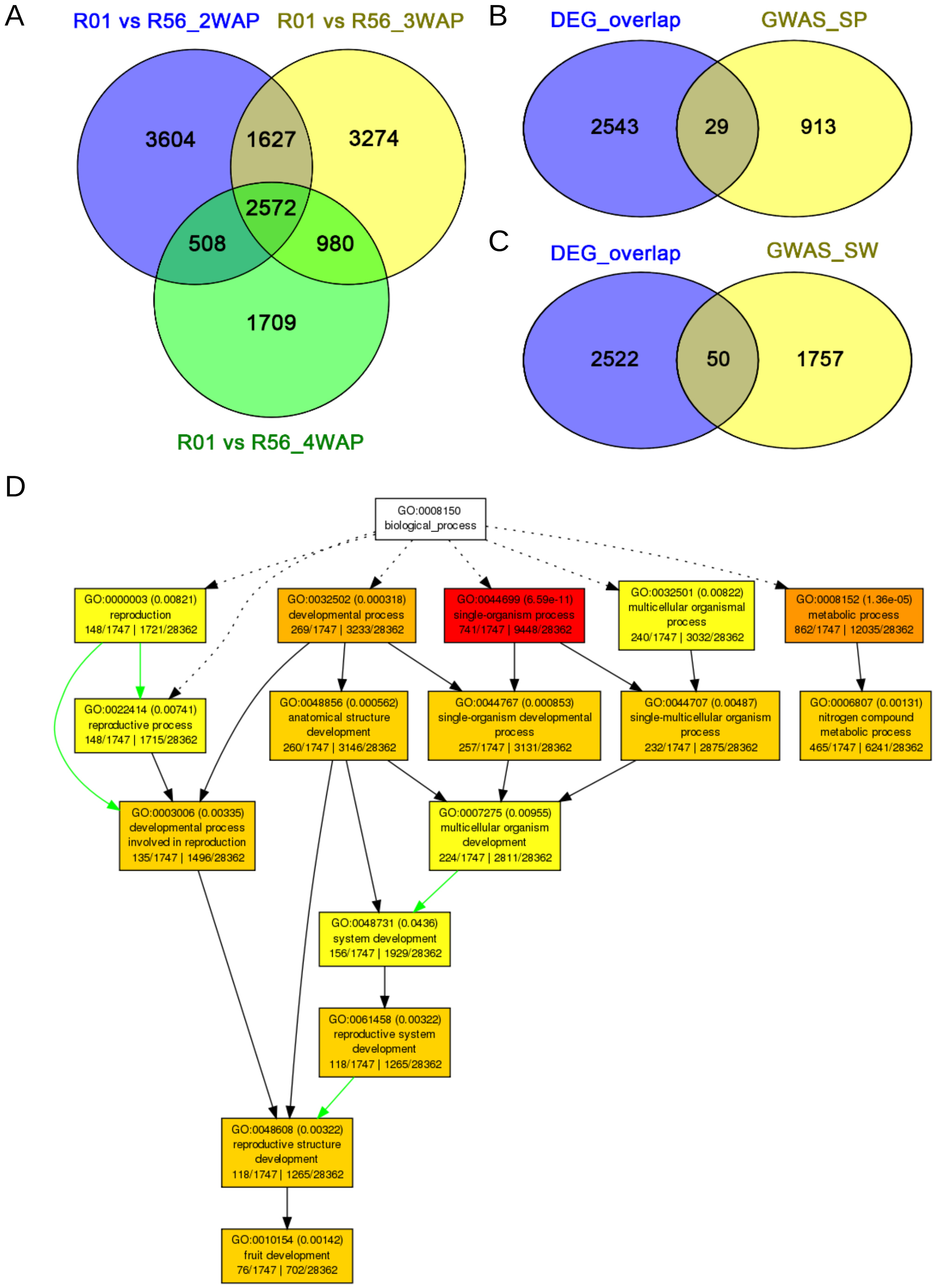

Figure S6 Comparative analysis of differentially expressed genes (DEGs) and GO enrichment analysis. (A) Venn diagram showing the overlap of DEGs in R01 vs R56_2WAP, R01 vs R56_3WAP, and R01 vs R56_4WAP. (B) Venn diagram showing the overlap of genes between 2572 DEGs_overlap and the candidate genes by multi-locus GWAS for seed per pod (SP). (C) Venn diagram showing the overlap of genes between 2572 DEGs_overlap and the candidate genes by multi-locus GWAS for 1000-seed weight (SW). (D) The GO enrichment analysis of the biological process (fruit development GO: 0010154) for 2572 DEGs. The depth of the color represents the degree of enrichment, with a darker color representing a higher degree of enrichment.

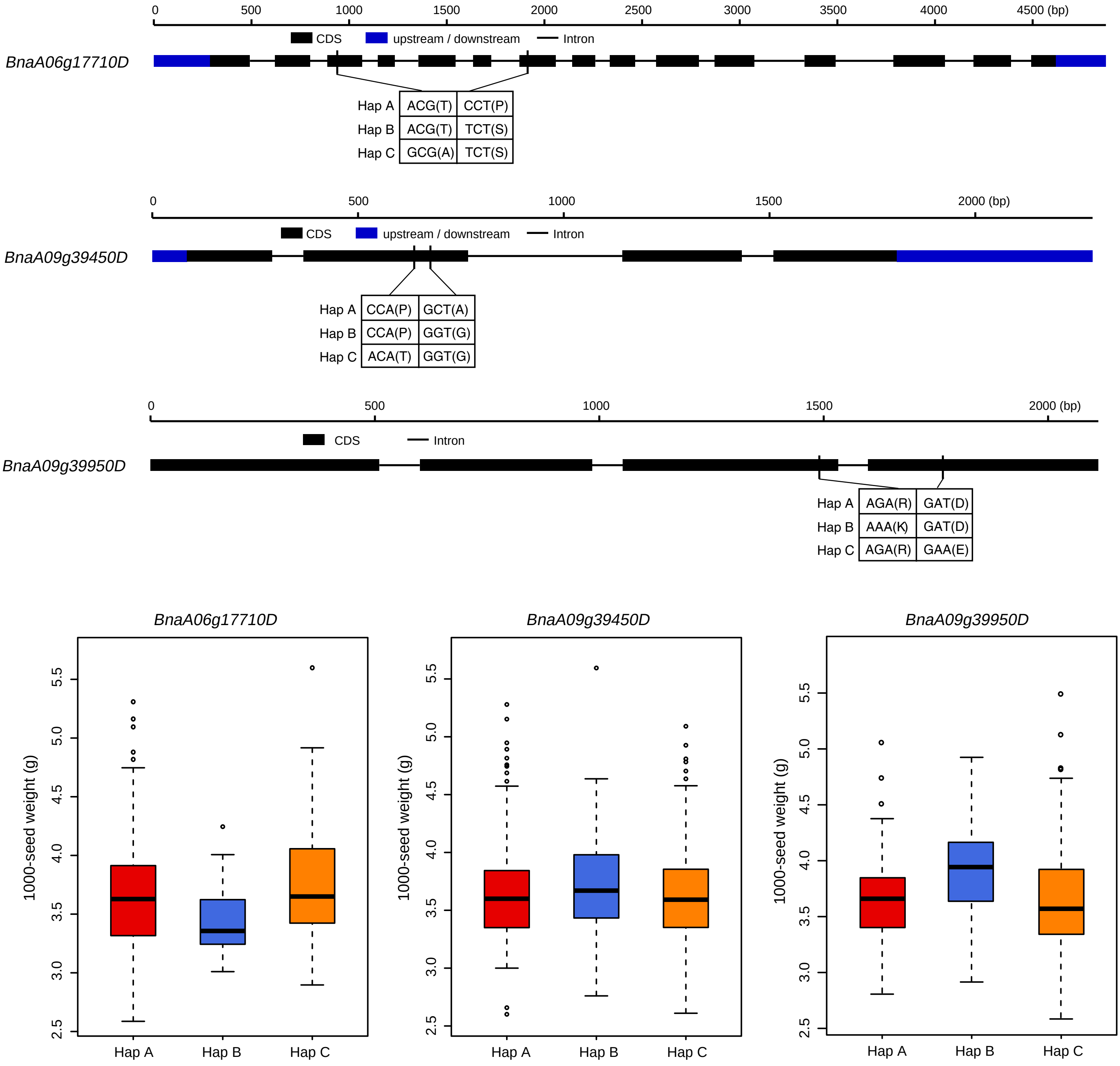

Figure S7 Haplotype analysis of the three candidate genes (*BnaA06g17710D*, *BnaA09g39450D* and *BnaA09g39950D*) for SW and their sequence variations in coding sites.

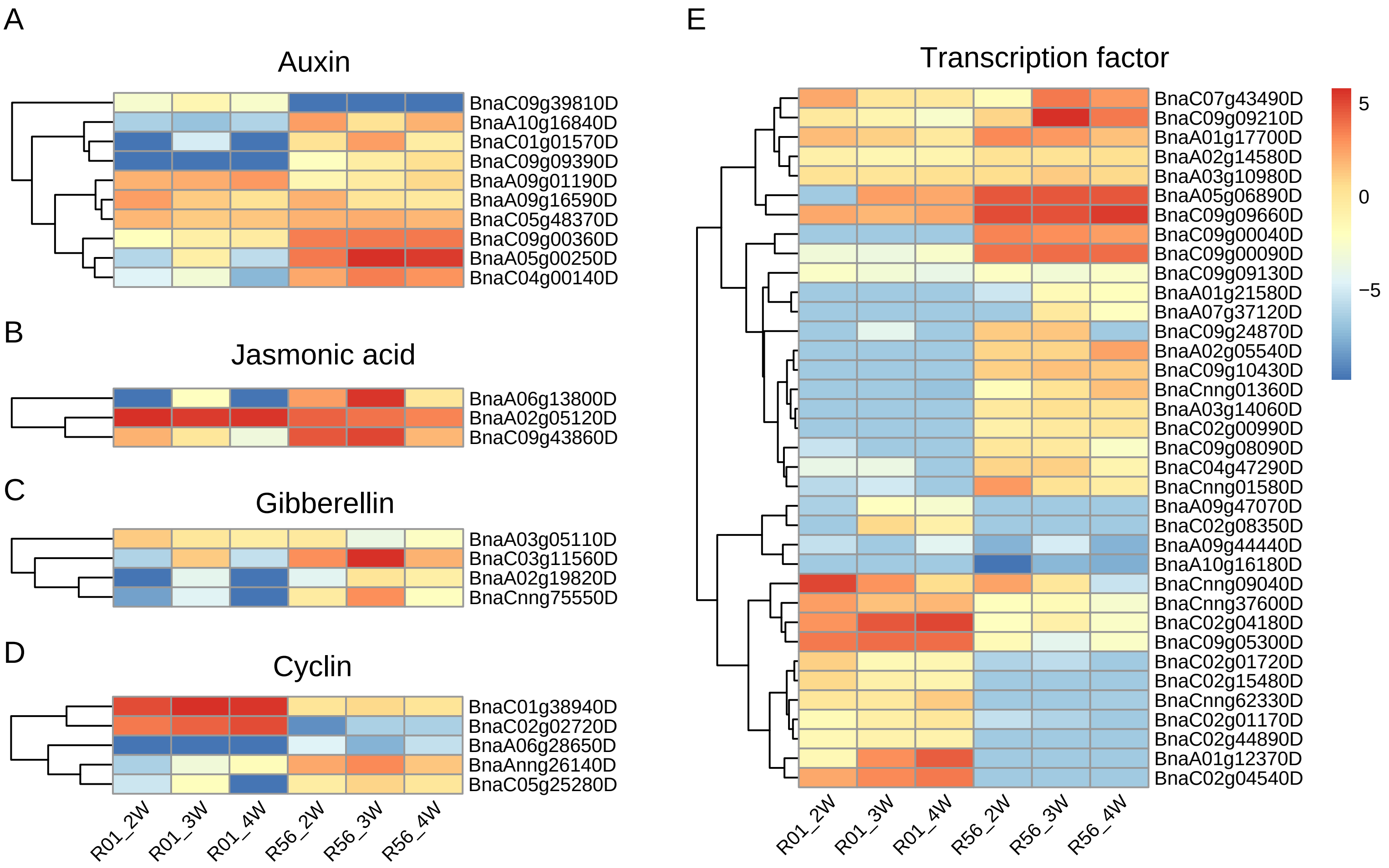

Figure S8 Heatmap of expression patterns of the differentially expressed genes (DEGs) between R01 and R56. All the heatmaps were based on log2 transformed FPKM values. (A) Auxin related genes. (B) Jasmonic acid related genes. (C) Gibberellin related genes. (D) Cyclin genes. (E) Transcription factors.
